# Self‐inflicted human bite followed by hand necrotizing fasciitis in an adult with Down syndrome

**DOI:** 10.1002/ccr3.2701

**Published:** 2020-02-05

**Authors:** Sho Komagoe, Shogo Azumi, Yuta Hasegawa

**Affiliations:** ^1^ Okayama Saiseikai General Hospital Okayama Japan

**Keywords:** delayed wound healing, Down syndrome, mental illness, necrotizing fasciitis, self‐harm bite

## Abstract

Adults with Down syndrome are more prone to develop intellectual, physical, and psychological disorders than their pediatric counterparts. It is pertinent to prevent the occurrence of severe complications in these patients. This case demonstrates the importance of support, regular follow‐up, and wound management in self‐care of adults with Down syndrome.

## Graphical abstract

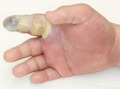

## 1 INTRODUCTION

Down syndrome is the most common genetic abnormality, and affected pediatric patients suffer higher intellectual disability and more health‐related issues than healthy children.^1^ Adults with Down syndrome also experience several clinical complications, with a higher prevalence of hypothyroidism, diabetes mellitus, obstructive sleep apnea, spinal cord compression, and cataracts.^2^ In addition, they are often affected by psychological disturbances such as dementia (including Alzheimer's disease), depression, and conduct disorder.^2^, ^3^ The point prevalence of mental illness of any type (excluding specific phobias), diagnosed using clinical criteria, in adults with Down syndrome was reportedly 23.7%.^4^ Alzheimer's disease has prevalence rates of 0%‐10%, 10%‐25%, 28%‐55%, and 30%‐75% in Down syndrome patients in their thirties, forties, fifties, and sixties, respectively.^2^ The duration of survival in these patients is reportedly affected by factors including the age at diagnosis of the mental illness, level of severity of the intellectual disability, living status, use of antidementia medication, and history of epilepsy.^5^ Moreover, Down syndrome is known to be associated with defective neutrophil chemotaxis, low humoral immune responses, zinc deficiency, and accelerated immunosenescence.^6^ These physiological and psychological disorders, along with intrinsic defects of the immune system, can result in various wound‐related complications, such as a late recognition of newly sustained injuries, prolonged wound‐healing, and chronic wound infections, in adults with Down syndrome.

## 2 CASE PRESENTATION

**Figure 1:**
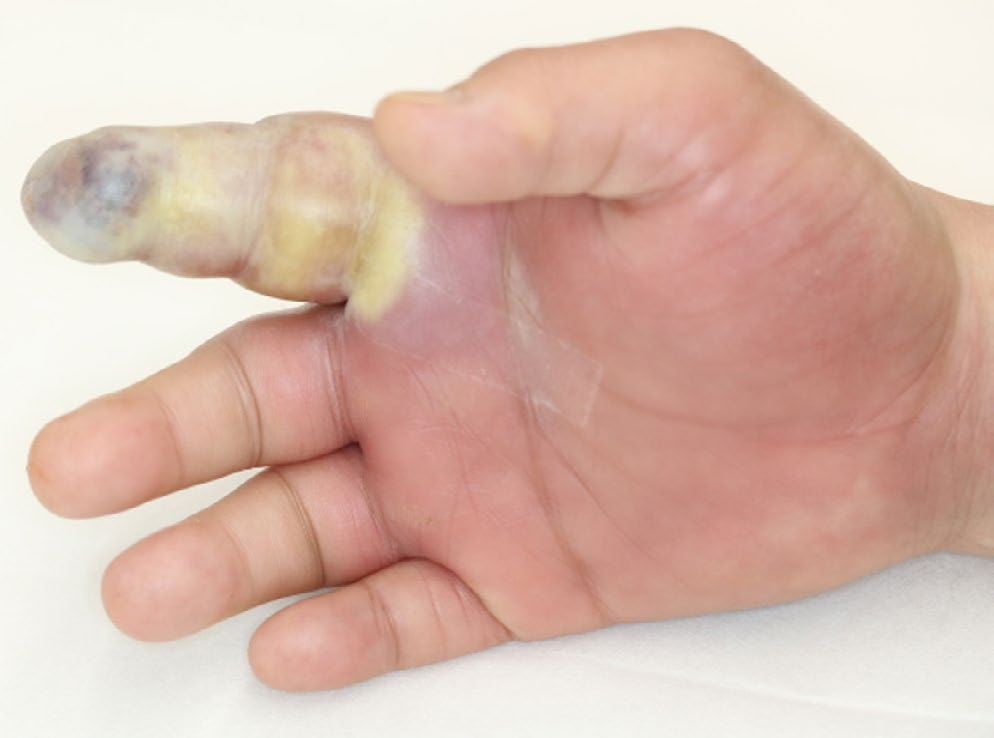
The patient's right hand shows diffuse swelling, especially involving the index finger

**Figure 2:**
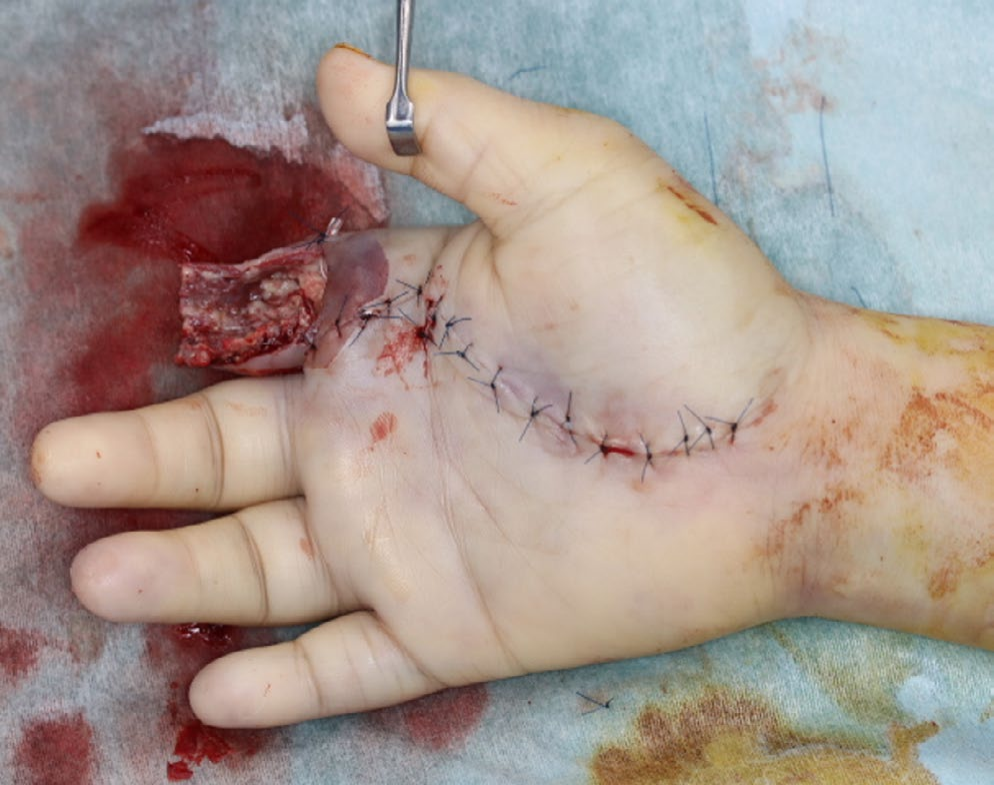
The right index finger was amputated, leaving the dorsal skin in situ

**Figure 3:**
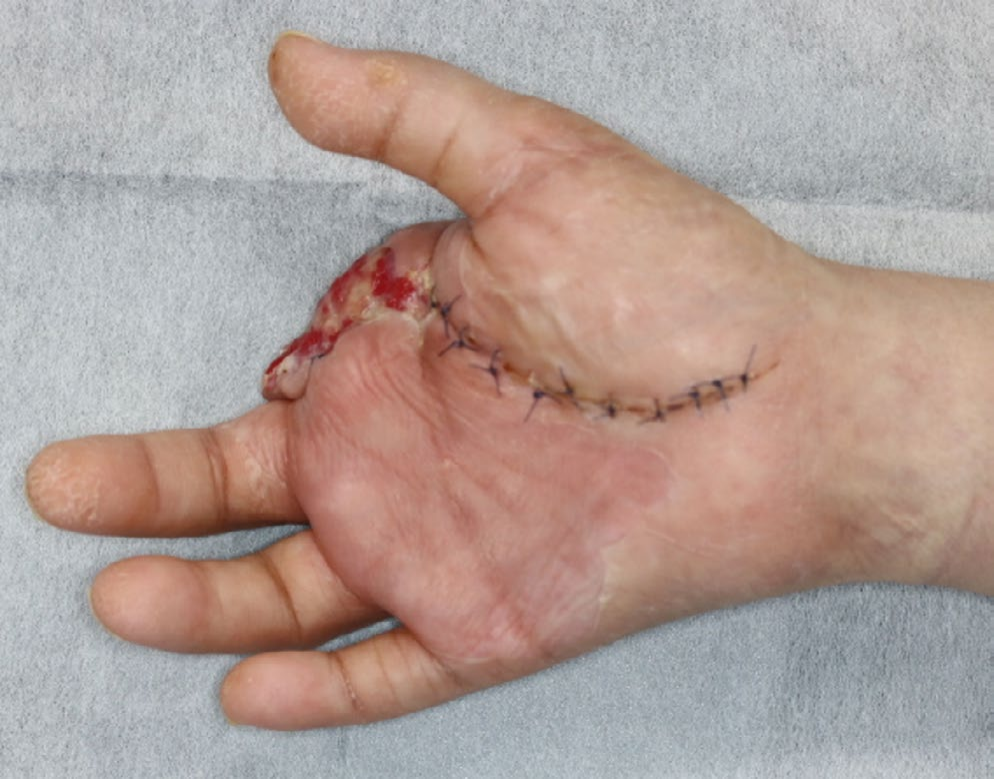
Good granulation tissue formation at the amputated site can be observed before the second surgery

**Figure 4:**
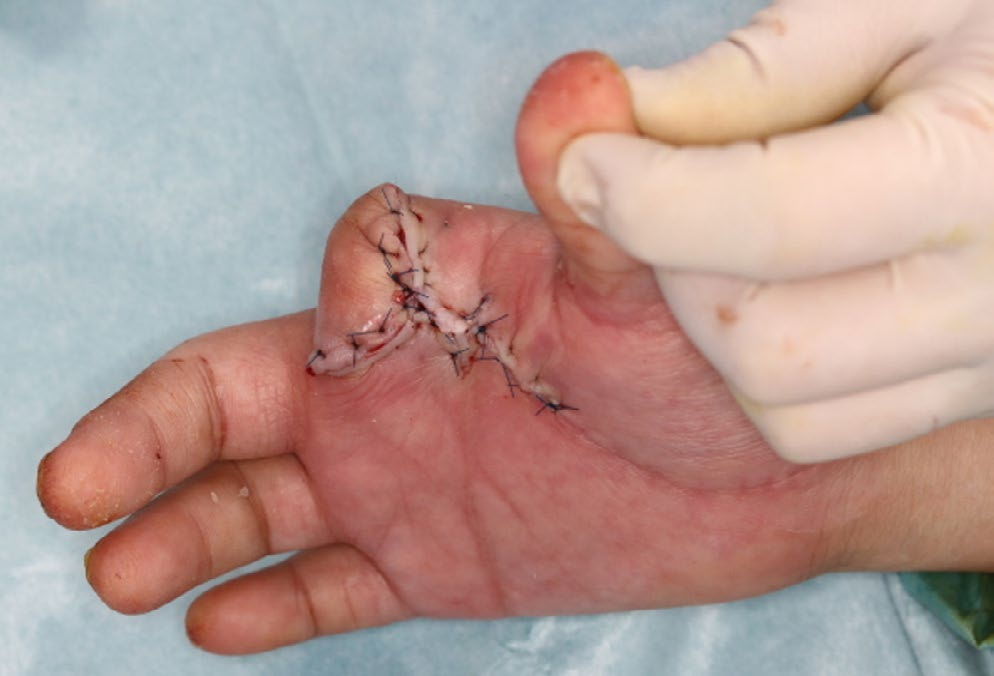
The wound was closed directly

A 47‐year‐old man was transferred to our hospital with complains of diffuse swelling and redness of his right hand. The patient had Down syndrome and had regularly visited a local psychiatrist for management of severe intellectual disability associated with the condition. The psychiatrist had started this patient on an atypical antipsychotic drug, olanzapine, for 1 year, to manage his excessive irritability, which had been reportedly controlled with the treatment. However, the patient had no history of regular visits to other doctors such as an internal medicine specialist. On examination, his right hand, especially the index finger, was observed to be severely swollen. The affected finger showed a subcutaneous accumulation of pus (Figure 1). The patient's habit of biting his right index finger was thought to be the cause of the infection. Blood examination results revealed severe inflammation (white blood cell count, 22 350/μL) and previously untreated diabetes mellitus (glycated hemoglobin level, 127 mmol/mol, normal <39 mmol/mol). We first performed emergent surgical debridement and amputation of the right index finger, following which the wound was left open to allow drainage (Figure 2). The wound was thoroughly irrigated, and intravenous antibiotics were administered daily for management of the infection. Furthermore, insulin was also administered to achieve glycemic control. The patient experienced severe intellectual disability, consequent to Down syndrome, and exhibited sporadic emotional outbursts during his hospitalization. Consequently, he was often uncooperative during the daily wound treatment, blood examination, and induction of anesthesia, which were exacting tasks. A new psychiatric assessment was performed during the hospitalization, and the patient's antipsychotic medication was changed from olanzapine to asenapine maleate, to prevent the hyperglycemia associated with the previous drug. His severe irritability was controlled relatively well with asenapine maleate during the period of hospitalization. The wound was closed directly during a second procedure, performed after 3 weeks (Figures 3, 4). The patient was discharged from the hospital 4 weeks after the first surgery, without any complications. He continues to visit a local physician regularly and has not experienced any other wound‐related issues or recurrence of the infection, 5 months after the first episode.

## 3 DISCUSSION

Human bites, similar to other mammalian bites, can easily cause deep‐tissue infections, especially affecting the hands, in patients with diabetes mellitus,^7^ such as in this case. Deeper, untreated bites can even cause severe morbidity (leading to amputation of the extremities) and consequent mortality.^8^, ^9^

This patient had a chronic habit of repeatedly biting his index finger, which may have resulted in a severe, polymicrobial infection that required an urgent amputation of the finger. Self‐inflicted human bite injuries have been reported in patients with mental disorders or those with risk factors for mental disorders. Dahlin et al have reported that stress, isolation, and loss of sensation associated with spinal cord injury lead to a development of habitual self‐biting and can consequently result in multiple finger amputation.^10^ In this case, diabetic neuropathy may have been a contributing factor in addition to the mental illness, allowing the patient to continue biting the injured digit.

To the best of our knowledge, this is the first case report of necrotizing fasciitis of the hand, caused by self‐inflicted human bites, in an adult with Down syndrome. We conclude that various clinical and pathophysiological factors characteristic of adult Down syndrome, combined with the repeated biting of the finger, together caused this serious infection. Furthermore, his intellectual disability and irritability made daily wound management a difficult task due to the patient's poor cooperation, which may have contributed to the delayed wound healing following the initial procedure. Fortunately, the patient's irritability was controlled during hospitalization, and he did not present with any other psychological manifestations, which would have further interfered with the daily wound‐care protocol. Upon reviewing the patient's history, clinical and psychological profiles, and after assessing for further risk of infection, we suggested that he would benefit from regular visits to both a psychiatrist and an internal medicine physician. Adults with Down syndrome are more likely to develop physical and psychiatric complications which can lead to difficult clinical presentations, similar to this case. Therefore, the treating doctors must be more attentive to providing proper early management of any wounds affecting such patients. It is also important to counsel the patient's family to direct the doctor's attention to any new injury, including bite wounds, which can be crucial in the preventing wound infection or halting its progression.

## 4 CONCLUSION

We described the case of an adult with Down syndrome, who presented to us with a self‐inflicted, severely infected, and complicated wound that required emergent management and demanding postoperative care for proper healing. We believe that reducing the incidence of similar instances is crucial. Therefore, we suggest a renewed focus on providing support and regular observation, with early wound management, as part of self‐care, in adults with Down syndrome.

## CONFLICT OF INTEREST

None declared.

## AUTHOR CONTRIBUTIONS

SK: wrote the paper and participated in the management of patient care. SA: guided treatment decisions and participated in the management of patient care. YH: participated in the management of patient care.

## INFORMED CONSENT

Consent was obtained from the patient's guardian for the use of the patient's medical information and images in this case report.
